# The global case fatality rate due to COVID-19 in hospitalized elderly patients by sex, year, gross domestic product, and continent: A systematic review, meta-analysis, and meta-regression

**DOI:** 10.1016/j.nmni.2022.101079

**Published:** 2023-01-04

**Authors:** Elham Davtalab Esmaeili, Hosein Azizi, Ehsan Sarbazi, Farzad Khodamoradi

**Affiliations:** a Student Research Committee, Tabriz University of Medical Sciences, Tabriz, Iran; b Road Traffic Injury Research Center, Tabriz University of Medical Sciences, Tabriz, Iran; c Research Center of Psychiatry and Behavioral Sciences, Tabriz University of Medical Sciences, Tabriz, Iran; d Department of Epidemiology and Biostatistics, School of Public Health, Tehran University of Medical Sciences, Tehran, Iran; e Department of Social Medicine, Faculty of Medicine, Ahvaz Jundishapur University of Medical Sciences, Ahvaz, Iran

**Keywords:** COVID-19, Elderly, Hospitalized, Meta-analysis, Mortality

## Abstract

**Background:**

Although elderly people are at a huge risk of mortality due to COVID-19, the Case Fatality Rate (CFR) in hospitalized elderly patients is poorly investigated. This meta-analysis and meta-regression aimed to generate pooled CFR due to COVID-19 in hospitalized elderly patients by sex, Gross Domestic Product (GDP), year, and continent and also to explain the potential source of the heterogeneity and variations in the pooled estimation of COVID-19 CFR.

**Methods:**

We systematically searched PubMed, Scopus, Web of Science, CINAHL, and Embase up to 31 July 2022. Eligibility assessment of records was performed independently in a blinded, standardized way by two reviewers. Meta-analysis and Meta-regression analysis were carried out to estimate pooled CFR and the potential sources of the heterogeneity.

**Results:**

The study included 5683 confirmed hospitalized elderly COVID-19 patients, 1809 deaths, and 19 original articles from 10 countries. The pooled estimate of the overall CFR, and by male and female sexes were 29%, 34%, and 24%, respectively. We found CFR was decreased by increasing female sex proportion, GDP, and year of publication. Multivariate meta-regression analysis indicated that the age and sex of patients, continent, GDP, and year of the publication together explained the majority of the heterogeneity and variations in the pooled estimate of the hospitalized elderly COVID-19 CFR.

**Conclusions:**

This review provided reliable pooled CFR measures for hospitalized elderly patients with COVID-19. Although COVID-19 fatality has decreased in hospitalized elderly patients over time, it is still high in hospitalized elderly patients and needs advanced treatment support.

## 1 Introduction

The ongoing novel severe acute respiratory syndrome coronavirus-2 (SARS-CoV-2); known as COVID-19; is triggering significant burden and fatality due to its infectious nature and the nonexistence of definitive management [1,2]. The COVID-19 infection imposed more than 474 million morbidities worldwide and about 6 million mortality as of March 24, 2022 [3].

Aging people are at a huge risk of morbidity and mortality with COVID- 19 infection because of age-related decline in immune function [4]. In the U.S.A, 80% of deceases reported have been in aging people over 65 years [5]. Currently, a population-based study revealed that the Case fatality Rate (CFR) due to COVID-19 is 24% in elderly people [6]. This study showed that CFR due to COVID-19 infection could increase by 70% in elderly people over the age of 85.

In the aging population, the majority of the published studies focused on risk factors for the mortality of elderly people with COVID-19 infection [[7], [8], [9], [10], [11]]. COVID-19 mortality measures are underreported in the general population due to a higher number of subclinical cases, underreported, and variations in the vital statistics [12]. The most accurate statistics for COVID-19 CFR are derived in the hospital setting, and for the general population, only excess deaths can be provided as an acceptable estimate for COVID-19 mortality and fatality [12,13].

However, inverse the general population, the hospitalized elderly COVID-19 CFR is poorly understood in the previous studies at the global level through meta-analysis design [14,15]. There are huge variations and heterogeneity between studies in the proportion of COVID-19 CFR due to the numerous variables including age, sex, socio-economic status, the hospital cares, continent, health system performance and policy, etc. [16,17]. Moreover, the hospitalized elderly COVID-19 CFR is a significant measure for the survival of the elderly with COVID-19 infection and the quality and appropriate COVID-19 case management. This is even though there has been a few studies focused on the hospitalized elderly COVID-19 CFR [14].

Gross Domestic Product (GDP), as a monetary measure of the market value of all the final goods and services produced in a specific time period by countries [18], was poorly understood in the COVID-19 CFR, especially in the systematic review and meta-analysis studies [19]. There is poor information about the hospitalized elderly COVID-19 mortality by GDP, age, sex, and continent [20].

The first aim of this meta-regression was to the pooled estimation of CFR due to COVID-19 in hospitalized elderly people by sex, GDP, age, and continent. The secondary goal of the study was to explore the potential sources and variations in the elderly COVID-19 CFR between studies and countries around the world.

## 2 Methods

### 2.1 Study design

This systematic review, meta-analysis, and meta-regression were conducted to estimate the global pooled CFR due to COVID-19 by sex, GDP per capita, year, and continent in hospitalized aging people (≥60 years old), and to explain the potential source of the heterogeneity and how COVID-19 CFR would is vary by sex, age, GDP, and continents.

### 2.2 Search strategy

We systematically searched the published literature in English from databases: PubMed, Web of Science, Scopus, CINAHL, and Embase, up to 31st July 2022. The study searched all records reporting CFR due to COVID-19 in hospitalized elderly patients around the world. Open Grey; WHO and CDC websites, and also google and google scholar free search were used to find grey literature. The reference lists of the retrieved articles and records were also screened with the purpose to identify other potential data sources.

The search conducted both free text words and medical subject headings (MeSH terms). The initial search terms were “COVID-19” OR “2019-CoV”, OR “2019 novel coronavirus” AND “fatality” OR “mortality” in the title and/or abstract. The final search used the relevant MeSH terms and text words related to COVID-19 case fatality in elderly people in conjunction with “elderly” OR “older” OR “aging” OR “geriatric” AND “fatality” OR “mortality” OR “death” AND “COVID-19” OR “2019-CoV”, OR “2019 novel coronavirus”.

### 2.3 Eligibility criteria

The inclusion criteria were all records evaluated the mortality or CFR due to COVID-19 in *hospitalized* aging people (60 years or over). We also excluded records that fulfilled at least one of the following criteria:

- Reviews, letters, commentaries, conference abstracts, editorials, and qualitative studies
- case reports and case series
- Records not evaluated the mortality or CFR in elderly patients (<60 years old)
- Records conducted in the general elderly people (not hospitalized) since the mortality and CFR of COVID-19 could differ between the general older people and hospitalized patients.
- records not related to CFR of the hospitalized COVID-19 patients
- Recodes evaluated the effectiveness of exposure including any drugs, vitamins, dietary, diseases, etc.
- Records with poor quality and/or high risk of bias and also studies reported incomplete data on CFR and the mortality and/or absolute numbers of COVID-19 survivors and non-survivors.

### 2.4 Outcomes

The primary outcome was pooled estimation of the total CFR due to COVID-19 in hospitalized elderly patients by sex, continent, GDP per capita (1000 USD), and the year of the publication. The CFR was defined [12] as the number (proportion) of people who died due to COVID-19 divided by the number of total confirmed COVID-19 cases multiplied by 100.

The secondary outcome was to explain the potential source of the heterogeneity to obtain variations in COVID-19 CFR.

### 2.5 Study selection and extraction

Two authors (HA and EDE) evaluated the eligibility of records independently in a blinded, standardized way. The records screening was conducted through title and abstract and then reviewers screened and selected relevant full-text papers. Discrepancies and disputes were resolved by consensus and the participation of one more author.

We extracted information including the year of publication, authors, study design, setting, country, mean age of total patients, mean age of survivors and non-survivors, male sex percentage, GDP per capita (world bank report, 2021), and the total CFR, and CFR by sex, year of the publication, and GDP per capita. The study included original research that was conducted in the setting of the hospital.

### 2.6 Quality and risk of bias assessment

The quality and risk of bias was evaluated based on Newcastle-Ottawa Scale [21]. The following parameters were considered for the quality assessment: sufficient sample size, sampling strategy (using random and unbiased sampling methods), appropriate data collection methods (for example no poor information, no admission of COVID-19 patients from a specific group, population, and specific region; as a selection bias), adequacy of response rate, inclusion/exclusion criteria, sample representativeness, and appropriate statistical analysis. The final scoring system comprised 11 criteria of rating different risk of bias elements for each eligible article out of 12 scores. Records were categorized into three levels of risk of bias: good; 9-12 points, fair; 5-8 points, and poor; <5 points (Table 1).

**Table 1:** Characteristics of studies included in the case fatality rate due to COVID-19 in hospitalized elderly patients

Authors	Year	Mean age			Country	GDP per capita (100 USD)	Male sex%	Design	COVID-19 patients (N)	Mortality (n)	Total CFR	Quality
		Total	Survivors	Non-survivors								
Bianchetti et al. [25]	2020	70.7	70	82	Italy	35.55	46.6	Retrospective	627	194	30.9	Good
Covino et al. [26]	2020	84	84	85	Italy	35.55	53.6	Retrospective	69	23	33.3	Good
De Smet et al. [27]	2020	85	84.5	88	Belgium	51.76	41.0	Retrospective	81	19	23.4	Fair
Heras et al. [9]	2020	85	86.5	86.1	Spain	30.11	38.0	Retrospective	100	20	20.0	Good
Lee et al. [8]	2020	72	71	77	Korea	34.75	45.0	Retrospective	98	20	20.0	Fair
Mendes et al. [28]	2020	86.5	86	87	Switzerland	93.45	43.0	Retrospective	235	76	32.3	Fair
Trecarichi et al. [29]	2020	80.5	78	85	Italy	35.55	57.1	Retrospective cohort	50	14	28.0	Fair
Zhou et al. [30]	2020	71.5	70.6	73.1	China	12.55	45.0	Retrospective	118	51	43.3	Fair
Sun et al. [31]	2020	69	67	72	China	12.55	54.5	Retrospective	244	121	49.6	fair
Mostaza et al. [32]	2020	85.2	85	86	Spain	30.11	54.7	Retrospective cohort	404	145	36.0	Good
Bavaro et al. [33]	2021	80	NR	NR	Italy	35.55	48.0	Retrospective cohort	206	56	27.2	Good
Becerra-Muñoz et al. [34]	2021	76	NR	NR	Spain	30.11	60.3	Retrospective	1520	541	35.6	Good
Covino et al. [35]	2021	85	84	87	Italy	35.55	47.0	prospective	239	77	32.2	Good
Fagard et al. [36]	2021	82	82	87	Belgium	51.76	52.4	Retrospective	105	14	13.3	Good
Qi Mei et al. [37]	2021	72	70	74	China	12.55	50.0	retrospective cohort	223	132	59.0	Good
Bakhshwin et al. [38]	2022	66	67	66	Saudi Arabia	23.58	55.0	retrospective	145	16	11.0	Fair
Asaduzzaman et al. [24]	2022	70	69.3	73.6	Bangladesh	2.50	64.5	Retrospective cohort	245	43	17.5	Fair
Maria Tam et al. [39]	2022	73	72	82	Hong Kong	49.66	52.4	Retrospective	101	17	16.8	Good
Ulugerger Avci et al. [40]	2022	74.4	74.4	76.7	Turkey	9.58	51.7	Retrospective	873	230	26.0	Good
**Total**	—	77.25	76.54	80.44	—	—	50.51	—	5683	1809	29.0	—

### 2.7 Analysis

Random-effects model [22] was used to estimate the pooled CFR and subgroup analysis by sex, continent, and GDP per capita with 95% confidence intervals (CIs). We carried out meta-regression analysis to explore the effect of age, sex, GDP per capita, continent and the year of the study published, when I^2^ was above 50%, suggesting heterogeneity [23]. Meta-regression analysis was used to explain the potential sources of the heterogeneity and variations in the elderly COVID-19 CFR between studies for the overall CFR, female sex CFR, and male sex CFR. All analysis were performed using STATA version 14.0 (Stata Corp, College Station, TX, USA).

## 3 Results

### 3.1 Characteristics of the included studies

**Fig. 1:**
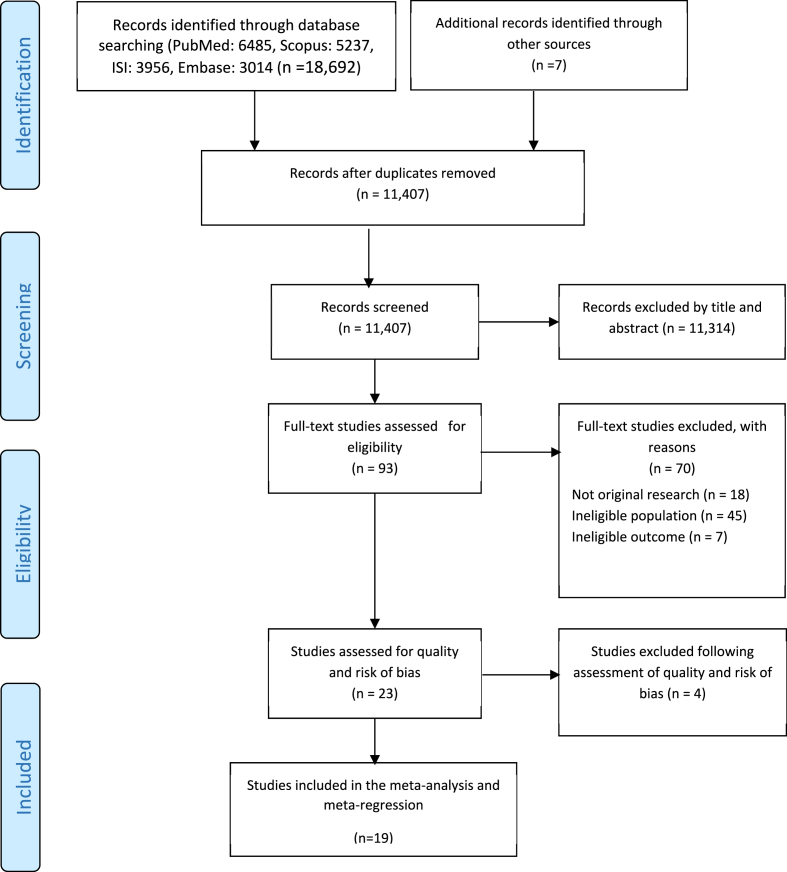
Search results and study selection and inclusion process.

A total of 18 699 records were retrieved in the study, and 7292 records were removed due to duplication. Of these, 11 314 were excluded because of irrelevant titles, abstracts, and texts. Then, 93 records were selected for the full-text review. Of these, 70 articles were removed due to not original research (n = 18), ineligible population or target group (n = 45), ineligible outcome (n = 7). Of these, 3 original studies were excluded due to poor quality and/or high risk of bias assessment. Finally, 19 articles were included in the meta-analysis and meta-regression (Fig. 1).

Overall, 5683 confirmed hospitalized COVID-19 patients and 1809 deceases were included in the study from 10 countries. The characteristics of the included studies and the result of the quality appraisal are shown in Table 1. All studies had been published between 2020 and 2022. The majority of the studies (5 articles) were conducted in Italy. The study setting and design of all the included studies were hospitals and retrospective, respectively. The mean age of the survivors, non-survivors, and the total elderly COVID-19 patients were 76.54, 80.44, and 77.25 years old, respectively. Regarding male sex proportion, the highest and lowest proportion of male sex participation was in Asaduzzaman et al. [24] and Heras et al. [9] studies, respectively. However, the overall male sex proportion in the included studies was almost 50.0% (Table 1).

### 3.2 Meta-analysis proportion of case fatality rate (CFR)

**Fig. 2:**
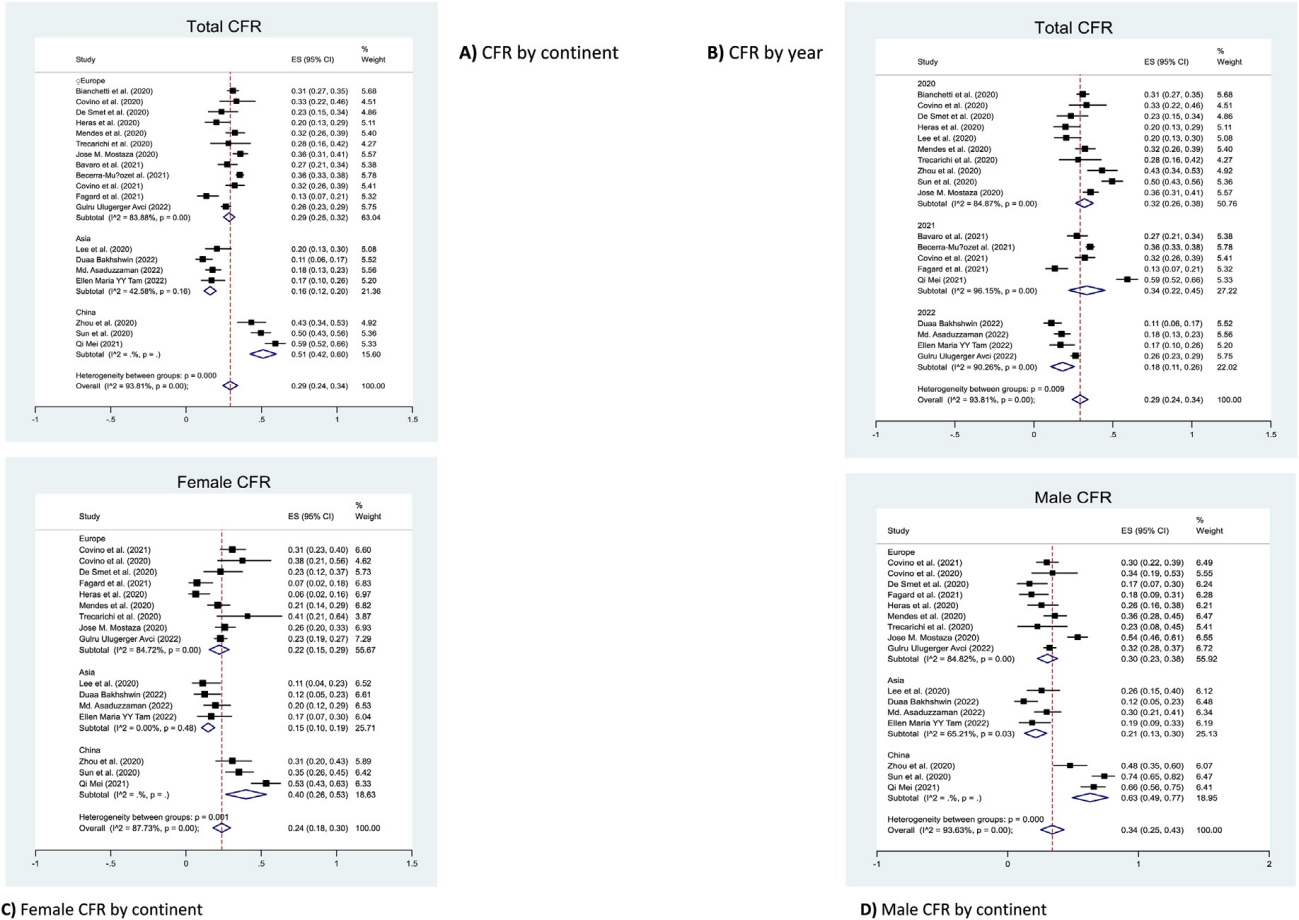
Sub-group meta-analysis proportion of hospitalized elderly COVID-19 case fatality rate by continent, year, and sex.

Fig. 2 showed that the sub-group meta-analysis proportion of hospitalized elderly COVID-19 case fatality rate by continent, year, and both sexes. The overall pooled proportion estimate of the elderly COVID-19 CFR was 29% (95% CI: 24-34%); I^2^ = 93.8%, p < 0.00; Subgroup analysis by continent demonstrated the pooled estimate of CFR 29% (95% CI: 25-32%); I^2^ = 83.8%, p < 0.00 for Europe; 16% (95% CI: 12-20%); I^2^ = 42.5%, p < 0.00 for Asia, and 51% (95% CI: 42-60%); I^2^ = 0.0%, p < 0.00 for China.

Concerning subgroup analysis of the proportion of CFR by year of publication, the pooled meta-analysis result using random effects for overall CFR was 32% (95% CI: 26-38%); I^2^ = 84.8%, p < 0.00 in 2020; 34% (95% CI: 22-45%); I^2^ = 96.1%, p < 0.00 in 2021, and 18% (95% CI: 11-26%); I^2^ = 0.0%, p < 0.00 in 2022.

Regarding female sex, the pooled estimate of the elderly COVID-19 CFR was 24 (95% CI: 18-30%); I^2^ = 87.7%, p < 0.00. While female sex CFR by continent was 22% (95% CI: 15-29%); I^2^ = 84.7%, p < 0.00 for Europe; 15% (95% CI: 10-19%); I^2^ = 0.0%, p < 0.00 for Asia, and 40% (95% CI: 26-53%); I^2^ = 0.0%, p < 0.00 for China.

Besides the pooled male sex CFR was 34 (95% CI: 25-43%); I^2^ = 93.6%, p < 0.00. However, female sex CFR by continent was 30% (95% CI: 23-38%); I^2^ = 84.8%, p < 0.00 for Europe; 21% (95% CI: 13-30%); I^2^ = 65.2%, p < 0.00 for Asia, and 63% (95% CI: 49-77%); I^2^ = 0.0%, p < 0.00 for China (Fig. 2).

**Fig. 3:**
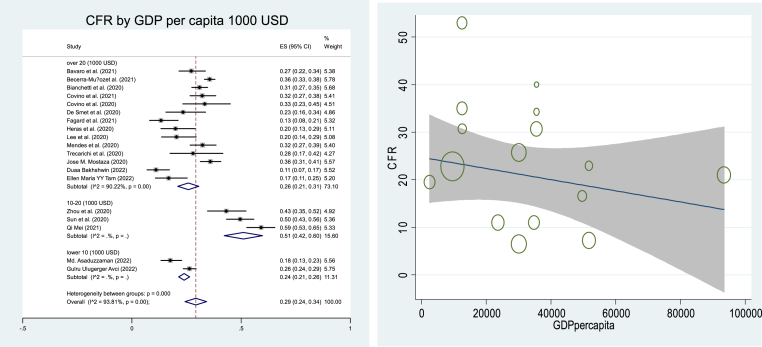
Sub-group meta-analysis proportion of hospitalized elderly COVID-19 case fatality rate by GDP per capita.

Concerning the overall CFR by GDP per capita (100 USD), the pooled estimate for the elderly COVID-19 CFR was 24% (95% CI: 21-26%); I^2^ = 0.0%, p < 0.00 for countries with GDP less than 10 (per 1000 USD); 51 (95% CI: 42-60%); I^2^ = 0.0%, p < 0.00 for countries with GDP 10-20 (per 1000 USD), and 26% (95% CI: 21-31%); I^2^ = 90.2%, p < 0.00 for countries with GDP over than 20 (per 1000 USD). We found the elderly COVID-19 CFR has been decreasing with a smooth slope by increasing GDP per capita. More details was presented in Fig. 3.

**Fig. 4:**
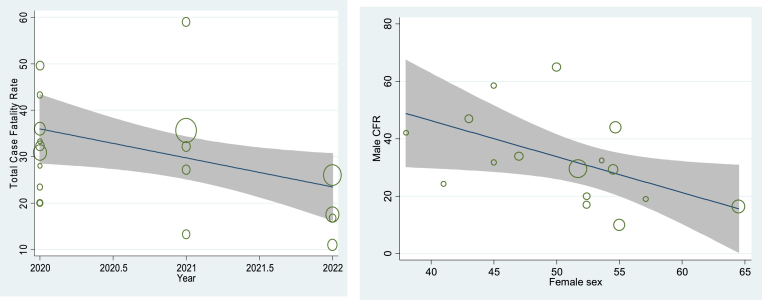
Trend of hospitalized elderly COVID-19 case fatality rate by year and female sex proportion.

Fig. 4 indicated that trend of hospitalized elderly COVID-19 case fatality rate by year and female sex proportion. We found the trend of the elderly COVID-19 case fatality rate decreased from 2020 to 2022. Moreover, there was a decreasing trend by increasing the ratio of female sex in the original studies.

### 3.3 Multivariate meta-regression

It is expecting a high heterogeneity (between studies variations) in the pooled estimation for proportional (prevalence) measures as in the current review. Therefore, multivariate meta-regression was conducted to explain variations in the hospitalized elderly COVID-19 overall CFR, and the analysis indicated that age, continent, GDP per capita, and year of the publication, in included studies covariates to be significant together and explained R^2^ = 70% of the between-study heterogeneity in the overall mortality (Table 2).

**Table 2:** Multivariable meta-regression analysis of study variables associated with the pooled estimate of the **overall CFR** in the elderly patient with COVID-19 (n = 19)

Variables	Coefficient	t	95% CI	P-value
Year of the publication	-5.79	-1.73	-12.8 – 1.28	0.102
GDP (per capita)	-0.02	-1.32	-0.004 – 0.001	0.112
Age (year)	5.26	1.99	0.96 – 0.12.08	0.068
Continent	10.77	2.24	0.30 – 21.02	0.045

Adjusted R-squared (R^2^) = 70.0%.

Table 3 indicates the results of multivariate meta-regression analysis to explain the potential heterogeneity in the pooled estimation of male sex CFR due to COVID-19 in the elderly. Age, continent, GDP per capita, and year of publication covariates to be significant together. These covariates together explained more than R^2^ = 80% of the heterogeneity in the pooled estimate of male-sex elderly COVID-19 CFR.

**Table 3:** Multivariable meta-regression analysis of study variables associated with the pooled estimate of the **male sex** CFR in the elderly patient with COVID-19 (n = 19)

Variables	Coefficient	t	95% CI	P-value
Year of the publication	-8.11	-1.92	-17.15 – 0.94	0.075
GDP (per capita)	-0.01	-0.85	-0.04 – 0.002	0.215
Age (year)	4.06	3.16	1.17 – 6.83	0.01
Continent	20.52	3.60	7.83 – 33.20	0.005

Adjusted R-squared (R^2^) = 80.43%.

Likewise, age, continent, GDP per capita, and year of the publication covariates together explained 61% of the total variance between studies in the pooled estimate of female sex elderly COVID-19 CFR (Table 4).

**Table 4:** Multivariable meta-regression analysis of study variables associated with the pooled estimate of the **female sex** CFR in the elderly patient with COVID-19 (n = 19)

Variables	Coefficient	t	95% CI	P-value
Year of the publication	-3.14	-0.93	-11.3 – 1.01	0.230
GDP (per capita)	-0.01	-0.94	-0.54 – 0.002	0.269
Age (year)	2.12	2.71	0.40 – 3.85	0.020
Continent	17.37	2.80	3.70 – 31.04	0.017

Adjusted R-squared (R^2^) = 61.0%.

Overall, based on multivariate meta-regression analysis; Table 2, Table 3, Table 4; 70%, 80%, and 61% of the heterogeneity in the pooled estimation was explained for overall CFR, male and female sex CFR, respectively, and the analysis decreased heterogeneity lower than 50% in all pooled measures. Continent and advanced age (years) increased the risk of the elderly COVID-19 CFR in the overall, male, and female sexes while male sex proportion, GDP per capita, and year of the publication had an inverse association with the elderly COVID-19 CFR.

## 4 Discussion

This meta-analysis and meta-regression consisted retrospective (case-control and cohort) studies demonstrating the pooled estimates of the elderly COVID-19 CFR by sex, continent, GDP per capita, and year of publication in hospitalized COVID-19 patients. Our analysis showed that COVID-19 CFR is decreased by increasing GDP per capita, female sex proportion, and year of publication.

The overall CFR, male and female sex CFRs in hospitalized elderly COVID-19 patients were 29%, 34%, and 24%, respectively. Some studies have shown that the mortality rate is lower in the general elderly population. For example in a study conducted in Brazil among general older adults, CFR was less than 12% [41]. Likewise, a population-based study in Iran indicated that CFR due to COVID-19 is 24% in the aging population [6]. However, the present study was conducted only among elderly in the hospital settings (hospitalized) where the mortality measures and COVID-19 statistics are more valid than in the general population [12].

Among generally admitted patients (all age groups) to the hospital, a systematic review found COVID-19 CFR is 17.1% [42]. Another meta-analysis in the initial period of COVID-19 pandemic indicated that the overall COVID-19 CFR is 10%; and hospitalized patients and patients who were admitted to the intensive care unit (ICU) were 13% and 37%, respectively [15]. Our study was performed among hospitalized elderly (60 years or over) COVID-19 patients demonstrating an overall CFR of 29%; and 32% in 2020; 34% in 2021; and 18% in 2022.

Multivariate meta-regression for the mortality model demonstrated that 70%, more than 80%, and 61% of study variations in pooled estimates for the overall CFR, male sex, and female sex CFR, respectively, could be explained by differences in the continent, age, GDP per capita, year of the publication and female sex proportion in the study samples. This finding is valuable in the factors affecting the global COVID-19 CFR patients. To our knowledge, this is the first meta-analysis and meta-regression to estimate the pooled hospitalized elderly COVID-19 CFR around the world.

We found COVID-19 CFR in the male sex is 10% higher than in the female sex, and the male sex is at a huge risk of mortality with COVID- 19 infection. As we showed in Fig. 4, with advanced female sex proportion in the original studies, COVID- 19 CFR has also decreased significantly. Previous findings and the included original studies in the current study confirmed that COVID-19 mortality is high in the male sex [[43], [44], [45], [46]].

Our analysis indicated that continent and advanced age were associated with an increased risk of CFR in both the male and female sexes. We found the highest and lowest pooled CFR was reported in China (51%) and Asia (16%). Likewise, the hospitalized elderly COVID-19 CFR was 29% in Europe. In line with our study, a meta-analysis found COVID-19 CFR in Europe and high-income countries showed an explosive increase compared with those in low-income countries, which can be interpreted as due to the under-reporting of mortality cases from COVID-19 [14]. Considering that China was the source and concentration of the emerging novel COVID-19, the high proportion of CFR may be justified while a lower risk of mortality in Asia deserves to investigate. Although, it seems that one of the main reasons is due to under-reporting and insufficient accuracy of the mortality and morbidity registration system.

Our analysis revealed that COVID-19 CFR is decreasing by advancing GDP per capita, female sex proportion, and year of publication. Fig. 3 shows that COVID-19 CFR decreased with a smooth slope by improving GDP measures. The measure of GDP is related to socio-economic status, development, income, and the quality of health and hospital services [47]. Ghayda RA et al. reported that CFR of COVID-19 is not a fixed or static value and it changed with socioeconomic status, time, population, and health systems efforts [14].

Regarding the year of publication, we observed a significant decrease in the elderly COVID-19 CFR from 2020 to 2022. Recent advances in the production of vaccines, drugs, and other treatments and hospital care could be the reasons for this downward trend by year. However, the proportion of CFR in hospitalized elderly people is high still, especially in China and Europe, although the accuracy of reporting and between-difference between countries should be taken into account.

### 4.1 Limitations

The current study is the first meta-analysis, and meta-regression analysis indicating the pooled CFR estimate of hospitalized elderly COVID-19 CFR by sex, continent, GDP per capita, and year of the publication, while our study had limitations. The main concern was the high heterogeneity and between studies variations in mortality measures from different countries with various hospital quality care and mortality reports. To solve this problem, first, we included only hospital setting studies, and also involved the potential effects of age, sex, continent, GDP of countries, and year using multivariate meta-regression analysis to explain the potential source of heterogeneity.

Second, to estimate CFR in hospitalized patients, concurrent cohort studies are appropriate. However, our systematic search found that there were very limited concurrent cohort studies and we also included case-control and retrospective cohort studies.

Third, the CFR in hospitalized patients with COVID-19 is affected by age, sex, socioeconomic status and GDP, underlying diseases, the type of mutation at the time of the outbreak, and medication history. Indeed, the current meta-analysis aimed to explore the impact of these variables in the pooled estimation of CFR and provide new hypotheses using sub-group and meta-regression analysis. Therefore, the current review not only provided the impact of these on CFR but it can be a departure point for future studies.

## 5 Conclusions

The COVID-19 10.13039/100004433CFR is high in hospitalized elderly patients and needs advanced treatment support. Based on meta-analysis, the pooled estimate CFR of COVID-19 is 29% overall, 34% in male, and 24% in female. The elderly COVID-19 CFR is higher in the male sex, continent (china), lower GDP per capita, year of the publication (in 2020); and meta-regression demonstrating these variables together explained the majority of the heterogeneity and variations in the pooled estimate of the hospitalized elderly COVID-19 CFR.

## Author statement

HA developed the original idea, developed the manuscript, interpreted and analyzed data, collected data, and drafted the manuscript. EDE, ES, and FKh contributed to the protocol and manuscript development, revising, editing, technical comments, and interpretation. All authors contributed to the manuscript development and/or made substantive suggestions for revision. All authors read and approved the final version of the manuscript.

## Funding

This study was performed with the financial support of Student Research Committee, 10.13039/501100004366Tabriz University of Medical Sciences. The authors also indicate that they did not have a financial relationship with the organization that sponsored the research.

## Ethical approval

The present study was derived from databases and no human interviews or samples were used. The study protocol was approved and reviewed by Ethics Committee, Tabriz University of Medical Sciences.

## Declaration of competing interest

The authors declare no competing interests regarding this study and its publication.

## References

[1] Girma D., Dejene H., Adugna L., Tesema M., Awol M. (2022). COVID-19 case fatality rate and factors contributing to mortality in Ethiopia: a systematic review of current evidence. Infection and Drug Resistance.

[2] Nakhlband A., Fakhari A., Azizi H. (2021). Interferon-alpha position in combating with COVID-19: a systematic review. Journal of Medical Virology.

[3] WHO (2022). https://covid19.who.int/.

[4] Kang S.-J., Jung S.I. (2020). Age-related morbidity and mortality among patients with COVID-19. Infection & Chemotherapy.

[5] Damayanthi H., Prabani K., Weerasekara I. (2021). Factors associated for mortality of older people with COVID 19: a systematic review and meta-analysis. Gerontology and Geriatric Medicine.

[6] Esmaeili E.D., Fakhari A., Naghili B., Khodamoradi F., Azizi H. (2022). Case fatality and mortality rates, socio-demographic profile, and clinical features of COVID-19 in the elderly population: a population-based registry study in Iran. Journal of Medical Virology.

[7] Dadras O., SeyedAlinaghi S., Karimi A., Shamsabadi A., Qaderi K., Ramezani M., Mirghaderi S.P., Mahdiabadi S., Vahedi F., Saeidi S. (2022). COVID-19 mortality and its predictors in the elderly: a systematic review. Health Science Reports.

[8] Lee J.Y., Kim H.A., Huh K., Hyun M., Rhee J.-Y., Jang S., Kim J.-Y., Peck K.R., Chang H.-H. (2020). Risk factors for mortality and respiratory support in elderly patients hospitalized with COVID-19 in Korea. Journal of Korean Medical Science.

[9] Heras E., Garibaldi P., Boix M., Valero O., Castillo J., Curbelo Y., Gonzalez E., Mendoza O., Anglada M., Miralles J.C. (2021). COVID-19 mortality risk factors in older people in a long-term care center. European Geriatric Medicine.

[10] Li G., Liu Y., Jing X., Wang Y., Miao M., Tao L., Zhou Z., Xie Y., Huang Y., Lei J. (2021). Mortality risk of COVID-19 in elderly males with comorbidities: a multi-country study. Aging (Albany NY).

[11] Lu Y., Jiao Y., Graham D.J., Wu Y., Wang J., Menis M., Chillarige Y., Wernecke M., Kelman J., Forshee R.A. (2022). Risk factors for COVID-19 deaths among elderly nursing home medicare beneficiaries in the prevaccine period. The Journal of Infectious Diseases.

[12] Azizi H., Esmaeili E., Fakhari A. (2020). Challenges and accurate estimates of mortality and case-fatality rates due to COVID-19. New Microbes and New Infections.

[13] Islam N., Shkolnikov V.M., Acosta R.J., Klimkin I., Kawachi I., Irizarry R.A., Alicandro G., Khunti K., Yates T., Jdanov D.A. (2021). Excess deaths associated with covid-19 pandemic in 2020: age and sex disaggregated time series analysis in 29 high income countries. Bmj.

[14] Abou Ghayda R., Lee K.H., Han Y.J., Ryu S., Hong S.H., Yoon S., Jeong G.H., Yang J.W., Lee H.J., Lee J. (2022). The global case fatality rate of coronavirus disease 2019 by continents and national income: a meta-analysis. Journal of Medical Virology.

[15] Alimohamadi Y., Tola H.H., Abbasi-Ghahramanloo A., Janani M., Sepandi M. (2021). Case fatality rate of COVID-19: a systematic review and meta-analysis. Journal of Preventive Medicine and Hygiene.

[16] Azizi H., Davtalab-Esmaeili E. (2020). Iranian first-line health care providers practice in COVID-19 outbreak. Iranian Journal of Public Health.

[17] Kim J., Hong K., Yum S., Gómez Gómez R.E., Jang J., Park S.H., Choe Y.J., Ryu S., Park D.W., Lee Y.S. (2021). Factors associated with the difference between the incidence and case-fatality ratio of coronavirus disease 2019 by country. Scientific Reports.

[18] Goossens Y., Mäkipää A., Schepelmann P., Van de Sand I., Kuhndt M., Herrndorf M. (2007). Alternative progress indicators to Gross Domestic Product (GDP) as a means towards sustainable development. Beyond GDP.

[19] Lippi G., Henry B.M., Mattiuzzi C., Bovo C. (2020). The death rate for COVID-19 is positively associated with gross domestic products. Acta Bio Medica: Atenei Parmensis.

[20] Cao Y., Hiyoshi A., Montgomery S. (2020). COVID-19 case-fatality rate and demographic and socioeconomic influencers: worldwide spatial regression analysis based on country-level data. BMJ Open.

[21] Wells G.A., Shea B., Da O’Connell, Peterson J., Welch V., Losos M., Tugwell P. (2000). Oxford.

[22] Grindem H., Mansournia M.A., Øiestad B.E., Ardern C.L. (2019). Was it a good idea to combine the studies? Why clinicians should care about heterogeneity when making decisions based on systematic reviews. British J Sports Med.

[23] Fathizadeh H., Milajerdi A., Reiner Ž., Amirani E., Asemi Z., Mansournia M.A., Hallajzadeh J. (2020). The effects of L-carnitine supplementation on indicators of inflammation and oxidative stress: a systematic review and meta-analysis of randomized controlled trials. Journal of Diabetes & Metabolic Disorders.

[24] Asaduzzaman M., Nazmul Alam Z., Zabed Jillul Bari M., Jahangir Alam M., Ranjan Chakraborty S., Ferdousi T. (2022).

[25] Bianchetti A., Rozzini R., Guerini F., Boffelli S., Ranieri P., Minelli G., Bianchetti L., Trabucchi M. (2020). Clinical presentation of COVID19 in dementia patients. The Journal of Nutrition, Health & Aging.

[26] Covino M., De Matteis G., Santoro M., Sabia L., Simeoni B., Candelli M., Ojetti V., Franceschi F. (2020). Clinical characteristics and prognostic factors in COVID-19 patients aged≥ 80 years. Geriatrics & Gerontology International.

[27] De Smet R., Mellaerts B., Vandewinckele H., Lybeert P., Frans E., Ombelet S., Lemahieu W., Symons R., Ho E., Frans J. (2020). Frailty and mortality in hospitalized older adults with COVID-19: retrospective observational study. Journal of the American Medical Directors Association.

[28] Mendes A., Serratrice C., Herrmann F.R., Genton L., Périvier S., Scheffler M., Fassier T., Huber P., Jacques M.-C., Prendki V. (2020). Predictors of in-hospital mortality in older patients with COVID-19: the COVIDAge study. Journal of the American Medical Directors Association.

[29] Trecarichi E.M., Mazzitelli M., Serapide F., Pelle M.C., Tassone B., Arrighi E., Perri G., Fusco P., Scaglione V., Davoli C. (2020). Clinical characteristics and predictors of mortality associated with COVID-19 in elderly patients from a long-term care facility. Scientific Reports.

[30] Zhou J., Huang L., Chen J., Yuan X., Shen Q., Dong S., Cheng B., Guo T.-M. (2020). Clinical features predicting mortality risk in older patients with COVID-19. Current Medical Research and Opinion.

[31] Sun H., Ning R., Tao Y., Yu C., Deng X., Zhao C., Meng S., Tang F., Xu D. (2020). Risk factors for mortality in 244 older adults with COVID-19 in Wuhan, China: a retrospective study. Journal of the American Geriatrics Society.

[32] Mostaza J.M., García-Iglesias F., González-Alegre T., Blanco F., Varas M., Hernández-Blanco C., Hontañón V., Jaras-Hernández M.J., Martínez-Prieto M., Menéndez-Saldaña A. (2020). Clinical course and prognostic factors of COVID-19 infection in an elderly hospitalized population. Archives of Gerontology and Geriatrics.

[33] Bavaro D., Diella L., Fabrizio C., Sulpasso R., Bottalico I., Calamo A., Santoro C., Brindicci G., Bruno G., Mastroianni A. (2021). Peculiar clinical presentation of COVID-19 and predictors of mortality in the elderly: a multicentre retrospective cohort study. International Journal of Infectious Diseases.

[34] Becerra-Muñoz V.M., Núñez-Gil I.J., Eid C.M., Garcia Aguado M., Romero R., Huang J., Mulet A., Ugo F., Rametta F., Liebetrau C. (2021). Clinical profile and predictors of in-hospital mortality among older patients hospitalised for COVID-19. Age and Ageing.

[35] Covino M., De Matteis G., Della Polla D.A., Santoro M., Burzo M.L., Torelli E., Simeoni B., Russo A., Sandroni C., Gasbarrini A. (2021). Predictors of in-hospital mortality AND death RISK STRATIFICATION among COVID-19 PATIENTS aged≥ 80 YEARs OLD. Archives of Gerontology and Geriatrics.

[36] Fagard K., Gielen E., Deschodt M., Devriendt E., Flamaing J. (2022). Risk factors for severe COVID-19 disease and death in patients aged 70 and over: a retrospective observational cohort study. Acta Clinica Belgica.

[37] Mei Q., Wang A.Y., Bryant A., Yang Y., Li M., Wang F., Du S., Kurts C., Wu P., Ma K. (2021). Survival factors and metabolic pathogenesis in elderly patients (≥ 65) with COVID-19: a multi-center study. Frontiers in Medicine.

[38] Bakhshwin D., Alotaibi M., Ali A.S., Althomali A., Alsuwat A., Alhamyani A., Alwathnani A., Alsaggaf S., Alrafiah A. (2022). Mortality predictors among COVID-19 elderly in Taif, Saudi Arabia. Infection and Drug Resistance.

[39] Tam E., Kwan Y., Ng Y., Yam P. (2022). Clinical course and mortality in older patients with COVID-19: a cluster-based study in Hong Kong. Hong Kong Med J.

[40] Ulugerger Avci G., Bektan Kanat B., Suzan V., Can G., Korkmazer B., Karaali R., Tabak F., Borekci S., Aygun G., Yavuzer H. (2022). Clinical outcomes of geriatric patients with COVID-19: review of one-year data. Aging Clinical and Experimental Research.

[41] de Souza C.D., de Arruda Magalhaes A.J., Lima A.J., Nunes D.N., de Fatima Machado Soares E., de Castro Silva L., Santos L.G., dos Santos Cardoso V.I., Nobre Y.V., do Carmo R.F. (2020). Clinical manifestations and factors associated with mortality from COVID-19 in older adults: retrospective population-based study with 9807 older Brazilian COVID-19 patients. Geriatrics & Gerontology International.

[42] Macedo A., Gonçalves N., Febra C. (2021). COVID-19 fatality rates in hospitalized patients: systematic review and meta-analysis. Annals of Epidemiology.

[43] Alkhouli M., Nanjundappa A., Annie F., Bates M.C., Bhatt D.L. (2020). Mayo clinic proceedings: 2020.

[44] Ramírez-Soto M.C., Arroyo-Hernández H., Ortega-Cáceres G. (2021). Sex differences in the incidence, mortality, and fatality of COVID-19 in Peru. PLoS One.

[45] Dehingia N., Raj A. (2021). Sex differences in COVID-19 case fatality: do we know enough?. The Lancet Global Health.

[46] Gebhard C., Regitz-Zagrosek V., Neuhauser H.K., Morgan R., Klein S.L. (2020). Impact of sex and gender on COVID-19 outcomes in Europe. Biology of Sex Differences.

[47] Maliszewska M., Mattoo A., Van Der Mensbrugghe D. (2020). The potential impact of COVID-19 on GDP and trade: a preliminary assessment. World Bank Policy Research Working Paper.

